# Development of a low-cost, user-customizable, high-speed camera

**DOI:** 10.1371/journal.pone.0232788

**Published:** 2020-05-08

**Authors:** Yamn Chalich, Avijit Mallick, Bhagwati Gupta, M. Jamal Deen

**Affiliations:** 1 Department of Electrical and Computer Engineering, McMaster University, Hamilton, ON, Canada; 2 Department of Biology, McMaster University, Hamilton, ON, Canada; 3 School of Biomedical Engineering, McMaster University, Hamilton, ON, Canada; University Hospital Eriangen at Friedrich-Alexander-University Erlangen-Numberg, GERMANY

## Abstract

High-speed imaging equipment can be an expensive investment, especially when certain applications require custom solutions. In this paper, we present a low-cost high-speed prototype camera built on a low-end Zynq-7000 System-on-Chip (SoC) platform and off-the-shelf components with the aim of removing the entry barrier into various high-speed imaging applications. The camera is standalone (does not require a host computer) and can achieve 211 frames per second (fps) at its maximum resolution of 1280x1024, and up to 2329 fps at a 256x256 resolution. With a current cost of only several hundred dollars and resource utilization of ~5%, the open-source design’s modularity and customizability allows users with sufficient hardware or programming experience to modify the camera to suit their needs, potentially driving the cost lower. This can be done by utilizing the large remaining programmable logic for custom image processing algorithms, creating user interface software on the CPU, attaching extensions through the peripheral Module connections, or creating custom carrier or daughter boards. The development and design of the camera is described and a figure-of-merit is presented to provide a value assessment of some available commercial high-speed cameras against which our camera is competitive. Finally, the camera was tested to record low frequency spatial vibration and was found to be useful in investigating phenotypes associated with aging in a leading animal model, the nematode (worm) *Caenorhabditis elegans*.

## Introduction

High-speed cameras have given us the ability to observe and analyze physical processes previously thought to be on timescales outside the realm of human comprehension. They are developed for machine vision applications [1] such as automatic inspection, used to conduct high-speed impact testing [2,3], and have allowed scientific research into the biomechanics of animals [4] and of humans in sports science [5], ballistics [6], and the broad field of fluid dynamics [7]. Unfortunately, commercial high-speed cameras can be a major investment. For instance, configurations of the Phantom–a popular high-speed video camera brand by Vision Research–can be priced $100,000 and up [8], creating a steep entry barrier. Now, high-speed imaging is seeing a widespread adoption in the mainstream consumer market, with start-ups offering more affordable alternatives [9,10] and flagship commercial phones such as the Samsung Galaxy S lineup recently boasting high-speed 960fps imaging as a key selling point [11]. Such devices can still be considered expensive with prices that hover in the thousands of dollars, and although mobile phone cameras have found interesting applications such as in low-cost microscopy [12], they are not particularly designed for some industrial and research applications due to their form factor, battery life and lack of configurability and processing capabilities. Upon investigating available commercial cameras, there appears to be a missing market of relatively very low-cost (low to mid hundreds of dollars) high-speed cameras that can offer framerates in the hundreds to low thousands at no less than a 256x256 resolution with enough storage for at least a few seconds of footage at no compression. To solve this with open-source hardware and off-the-shelf electronics would help researchers and enthusiasts facilitate their development of such a camera. The minor associated development costs can result in a large return on investment of hundreds to thousands of percent [13].

We provide a working prototype solution built upon a Zynq SoC (System-on-Chip) containing a CPU and FPGA (Field-Programmable Gate Array) with a CMOS (Complementary Metal-Oxide-Semiconductor) image sensor that can lead the way towards the development of these low-cost high-speed cameras. Similar architectures have been investigated before [14], but given current hardware advancements, our use and organization of off-the-shelf components with a custom FPGA implementation provide a framework for a build-your-own style, standalone (no host computer required) camera that is competitive in the market in terms of cost and functionality. Some of the most inexpensive and directly comparable cameras to this would be those in the machine vision industry. However, they are typically not standalone and depend on device link throughput to a host computer, thus limiting their framerates. Our approach is similar to the approach of some start-ups such as Kron Technologies Inc. [9] and The Slow Motion Camera Company Limited [10] that create high-speed cameras in the low to mid thousands of dollars using custom architectures and efficient optimizations. For instance, Kron Technologies Inc. uses a ten-year-old ARM Cortex A8 CPU for the user interface, and a $35 Lattice FPGA to grab data from its sensor at high speeds where an analogous FPGA in a Phantom camera can cost thousands of dollars [15].

In fact, the performance benefits of FPGAs play a large role in the implementation and optimization of the high-speed designs required to grab and process data from an image sensor. FPGAs run at clock speeds orders of magnitude slower than their embedded processor equivalents, but their high degree of parallelization can create spatially-oriented circuits that allow application speedups in both latency and throughput compared to a software implementation [16]. Since FPGA designs also implement the data and control paths, they can offer fully deterministic performance, avoiding the fetch and decode pipeline that can severely limit software execution on a processor [17,18]. Furthermore, they have become important in fields such as deep learning due to their efficiency as hardware accelerators, outperforming GPUs in performance per Watt for some important subroutines [19]. Despite their excellent potential performance and versatility, FPGAs have been referred to as a “specialist architecture” [19] and coding them in hardware descriptive languages (HDLs) is “strenuous” [17]. Compared to how software programming has evolved, hardware programming on FPGAs is still very low level in terms of abstraction, more difficult to debug, and uses more complex EDA tools among other things [18], leading to a lack of hardware programming literacy among scientists and engineers.

With some FPGA programming experience and standard commodity parts, one can modify and expand on our high-speed camera prototype, which contains plenty of resources left for advanced processing capabilities. Having a cost similar to machine vision CMOS cameras of several hundreds of dollars (USD), it also includes the added functionality and benefits usually found in more expensive standalone cameras. For instance, the Kron Technologies Inc. Chronos 1.4 camera was recently independently tested as a potential low-cost, high-speed, scientific imaging camera [20] and was reported to have beneficial features like external triggering and binary data output not found in other low-cost consumer cameras, but which our camera is also capable of. It was also found to be well suited for high resolution forward-scattering and in-line imaging like bright-field microscopy, which similarly makes biology and microscopy a key application area for our camera as well. The field of microscopy has seen research in similar spirit to ours with a low-cost, open-source multi-fluorescence imaging system [21], and our solution can help accelerate or remove unneeded complexity in high-speed designs, such as in [22] which required a custom-built sensor, DAQ card, and host computer for the high-speed imaging of the blood flow and heartbeat of live Daphnia.

In this paper, the camera design implemented using a Zynq SoC Microzed development board, an FMC carrier board, and a custom printed circuit board (PCB) for the image sensor is described, serving as a framework by which others can develop custom solutions. Then, a figure-of-merit (FoM) is also created to analyze and compare the performance of some available standalone and machine vision high-speed cameras with our own to show the value proposition of our design. Next, we explore the camera’s customizability in terms of both hardware and software to suit various research conditions. Lastly, we demonstrate the camera’s high-speed capabilities by recording low frequency spatial vibration and show that it can outperform commercial entry-grade cameras used routinely in biological research and teaching.

## Materials and methods

### Component selection

Being one of the costliest components in a camera, proper selection of an image sensor for this market area is crucial. Although CCD (Charge-Coupled Device) image sensors can offer a better signal-to-noise ratio and dynamic range suitable for still images, CMOS image sensors have managed to significantly catch up, all while offering a variety of benefits over them as a result of sharing a fabrication procedure with integrated circuits. CMOS image sensors offer lower power, in-pixel charge-to-voltage conversion for high-speed readout, on-chip peripheral components such as PLLs (Phase-Locked Loops) and ADCs (Analog-to-Digital Converters) for complete system integration, and all at a lower cost [23]. For our design, the PYTHON 1300 CMOS image sensor was chosen. A comparison of CMOS image sensors typically used in some low-cost machine visions systems [24] shows that sensors such as the SONY IMX lineup can have better sensitivity and noise performance, however, ease of attainability and development, and high framerates were the deciding factors in selecting the PYTHON 1300. It was the most readily available through several distributors in single quantities at a relatively low and affordable price, has an extensive and detailed datasheet [25] and very competitive high-speed performance allowing us to comfortably reach near or above 1000 fps at good resolutions. It has an array size of 1280x1024 pixels (1.3 MP) and is specified to achieve 210 fps at full resolution with up to 2235 fps at a 256x256 region of interest (ROI) selection. The sensor is configurable through a serial peripheral interface (SPI) and outputs 6 low-voltage differential signaling (LVDS) pairs (a 360 MHz clock, 1 sync channel, and 4 data channels). The monochrome version (peak quantum efficiency of 56% at 550 nm) was chosen over the Bayer colour version because this allows the Bayer pattern demosaic algorithm to be skipped and the lack of RGB filters enhances light sensitivity by allowing up to 3x more light to hit each pixel. The improved sensitivity allows the monochrome sensor to be better suited for scientific research, even though the sensor itself is only marketed for machine vision, motion monitoring, security, and 2D barcode scanning.

Next, we looked to use off-the-shelf components to build a camera in a standalone form factor with the memory and hardware necessary to maximize the capabilities of this sensor. The MicroZed development board with the Zynq-7000 SoC family [26] offered a great option in this regard for being a cost-optimized solution containing both programmable logic (PL) (based on Artix-7 FPGA) and a processing system (PS) (dual-core ARM Cortex-A9 processor). Of the low-end models, the more expensive Zynq 7020 SoC was selected due to the increased on-chip resources to speed up the debugging process, but due to the very low (~5%) resource usage of our design, even the cheapest model chip could work in an optimized implementation. Although the design is primarily PL-based, the inclusion of the PS allowed for a simple method to transfer stored frames to an SD Card for permanent storage and also helps provide a wider range of future design considerations. Of the Zynq-based development boards, the MicroZed was selected due to its versatile MicroHeader expansion, proven capabilities to handle the high-speed LVDS signals required to interface with the image sensor, and above all, its large 1 GB DDR3 memory for acting as a buffer for frames captured at high framerates.

**Fig 1:**
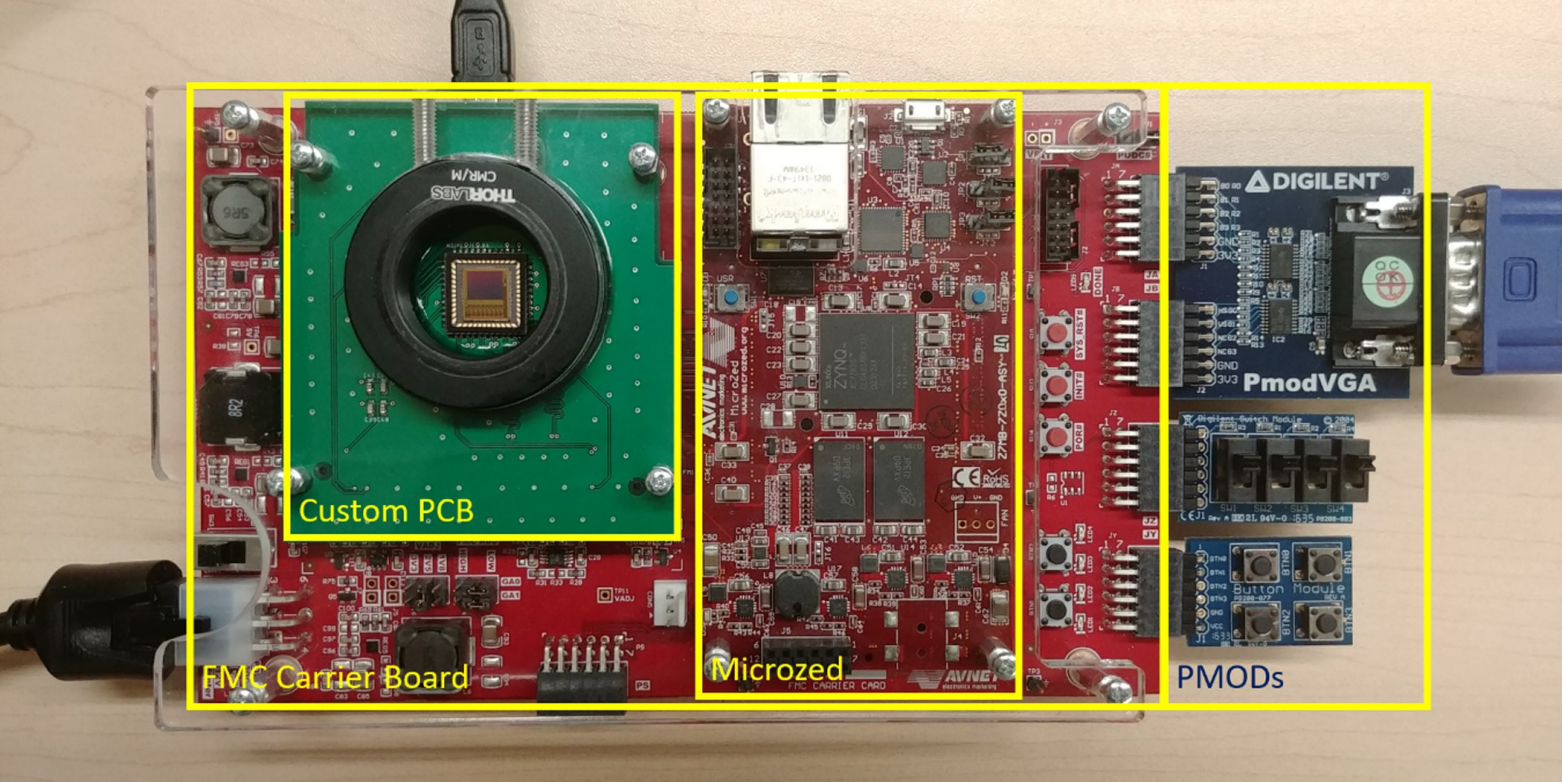
Full camera prototype containing the Microzed, FMC carrier board, custom camera sensor module and PMOD extensions. An acrylic protective cover and c-mount lens provide a temporary solution for safe mounting.

In order to access the necessary I/O banks in the PL through the MicroHeaders, a carrier board was used. There exists a MicroZed embedded vision development kit that includes a PYTHON 1300 module, but the overall cost of the evaluation kit goes above $1000 USD, with the camera module alone being half that cost [27]. The kit also requires licensed IP and is limited in its customizability and standalone capabilities. We decided on choosing the cheaper and more versatile FMC (FPGA Mezzanine Card) carrier board and designed our own camera PCB at a fraction of the cost. The FMC connector can support up to 36 LVDS pairs making this configuration capable of handling much more than the 6 pairs we require if a more capable (and more expensive) sensor is needed. Another benefit of the FMC carrier board was its multi-purpose Peripheral MODule (PMOD) headers which was used to expand the functionality of the camera through pushbuttons, switches, and a VGA output (by Digilent Inc.) to give the camera its standalone quality. A custom-cut acrylic cover protected the system and a C-Mount-threaded lens mount was incorporated to allow mounting onto microscope systems or attach lenses, but it is recommended that future designs use a 3D-printed housing solution to further lower costs. A spacer was also included to lift the C-mount to provide better focus with attached lenses. The flat, open design of our prototype also meant it required no cooling. Fig 1 shows a picture of the assembled system with the described components and PMOD attachments.

### Image sensor PCB design

The design required a custom PCB for the image sensor that could supply power to the sensor and route its I/O to the FMC connector pins appropriately. Two supply voltages of 3.3 V and 1.8 V were required, as well as another pixel supply voltage of 3.3 V. The tolerances for each differ and range from 1.5% to 5%, thus requiring separate high-quality voltage regulators to meet the specs when used in conjunction with a general-purpose 5V micro-USB power supply. Although the FMC connector can supply a 12 V power line, the PCB was initially designed to be powered independently for testing and possibly act as a custom carrier board for the MicroZed in the future, so the external power was kept in the design.

**Fig 2:**
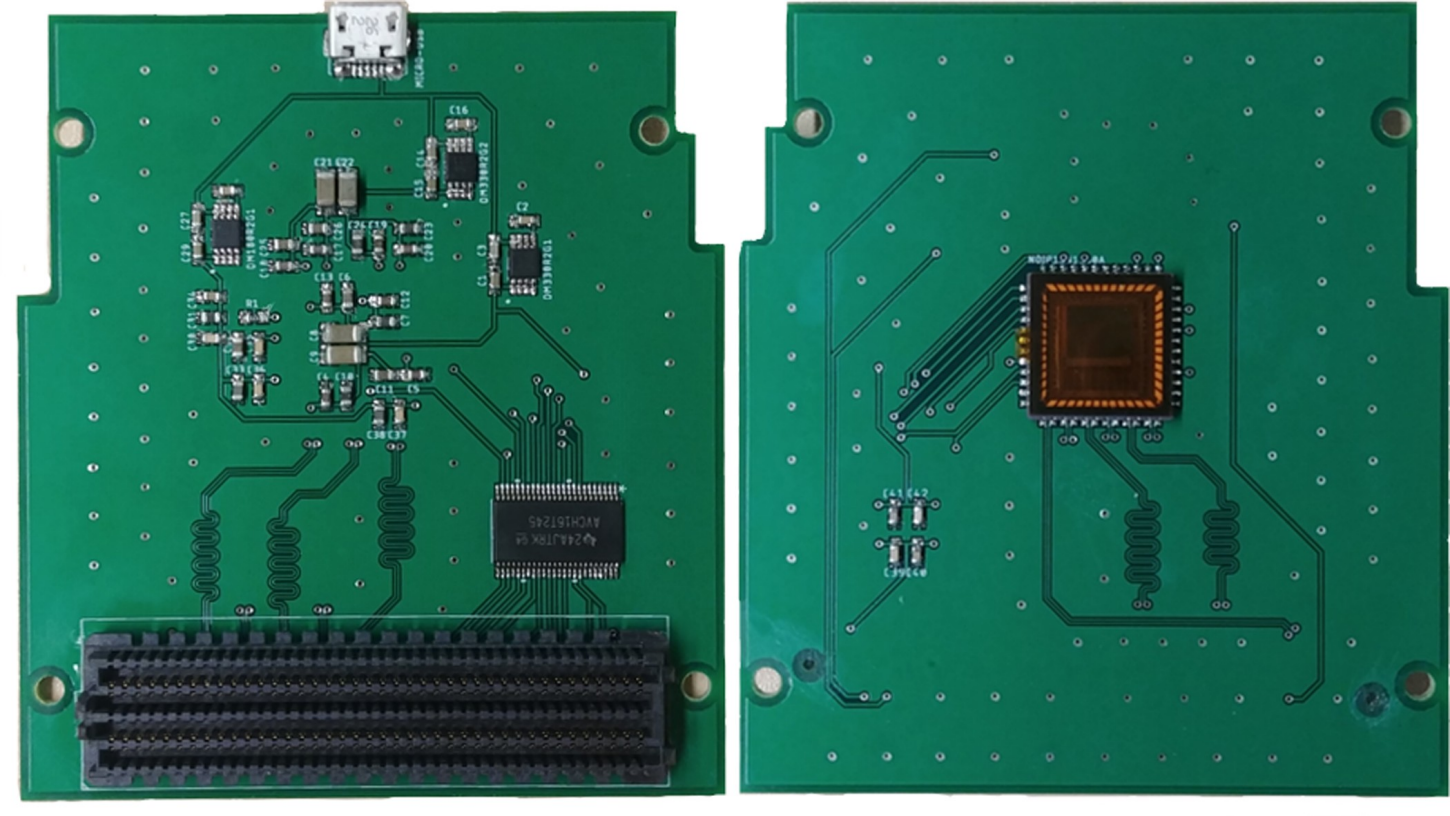
PYTHON 1300 camera sensor PCB design with an FMC connector.

The LVDS output of the sensor has a common mode voltage of 1.25 V for transferring pixel, timing and sync data out, while the single-ended signals operate at 3.3 V for sensor configuration, monitoring, and triggering. The LVDS standard required setting the voltage level of the carrier board, and thus the I/O bank used, to 2.5 V to act as a proper receiver. Furthermore, the differential traces on the PCB were designed to be length matched through meandering and set to 100 Ω differential impedance. With the Zynq chip’s I/O bank limited to operating at 2.5 V, a voltage level shifter was required to translate the single ended signals between 2.5 and 3.3 V. To better isolate the LVDS lines and improve RF performance, a 4-layer board was used where the 2 internal layers were grounded and via stitching connected the 4 ground planes together. The majority of the components were placed on the back of the PCB so as to minimize the obstruction to any housing solution, such as with the C-mount used. A close-up of the front and back of the PCB is presented in Fig 2.

### HDL design

**Fig 3:**
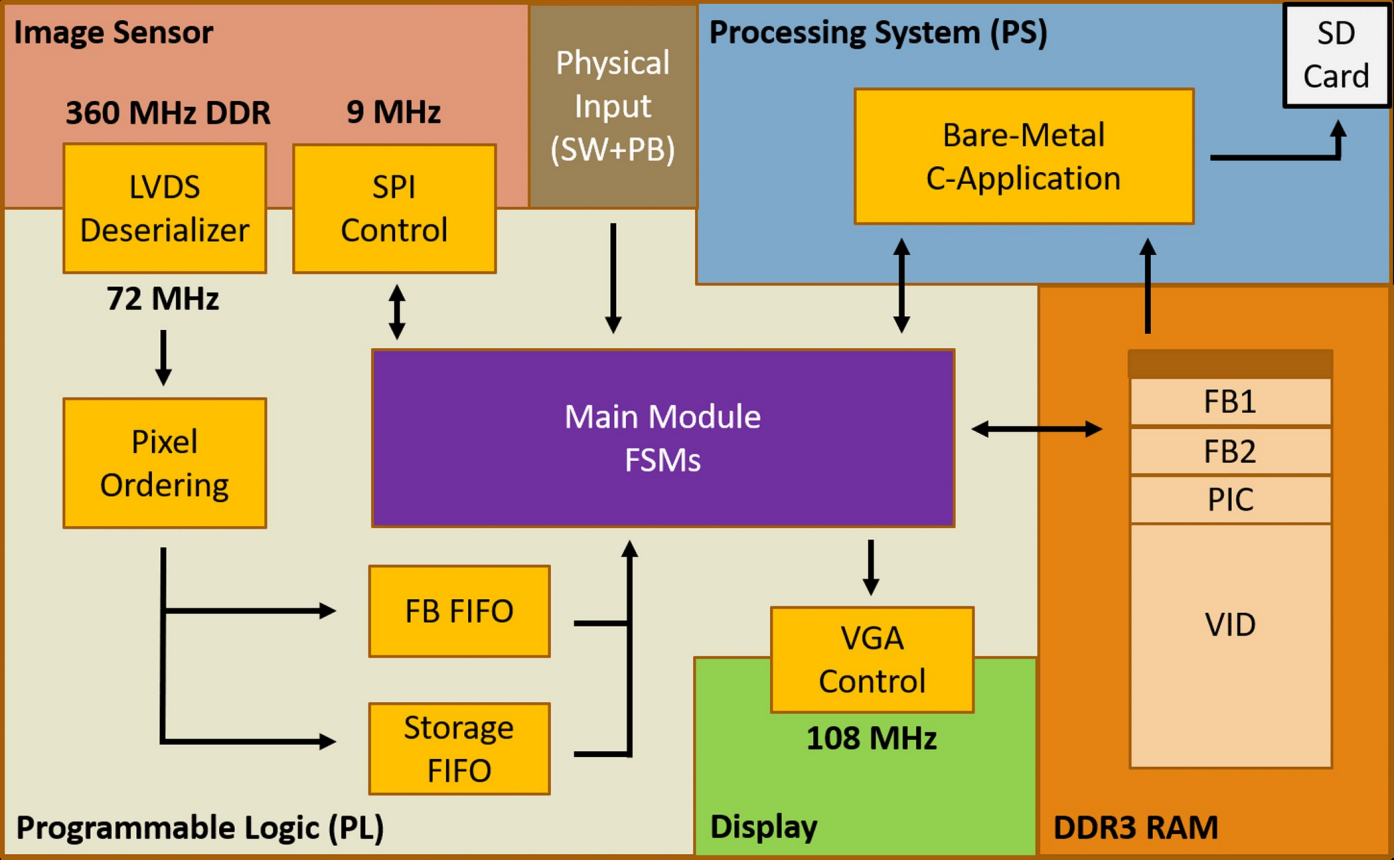
Design flow including camera configuration, pixel storage, and display output.

From here, our codebase (written primarily in SystemVerilog) must power the camera sensor correctly, configure it through the SPI protocol, align and process the LVDS input, and finally store or display the captured frames, all operated using pushbuttons (PB) and switches (SW). The flow of this design is displayed in Fig 3 and is developed using the free Vivado WebPACK version 2017.4.1 (in which Zynq-7000 SoCs are supported devices).

The LVDS data channels of the image sensor transmitted serially at a double data rate (DDR) of 720 Mbps over the nominal 360 MHz clock sent through the clock channel. A Xilinx reference design (XAPP1017) was used and slightly modified to receive and deserialize the data, synced to the differential clock channel from the sensor. The pixel data was then organized in order due to the unique readout order of the sensor and then sent simultaneously to two different FIFOs (first-in first-out) before being processed and stored into RAM. FIFOs allow their input and output ports to operate at different clock speeds making them useful for designs that cross clock domains. One FIFO facilitates the storage of frames in frame buffers (FB) for real-time display on a monitor while the other captures all incoming frames for picture or video storage.

Switching of the FBs is automated based on the completion of the display of one frame and the buffering of the next. When one FB is being filled with pixel data, the other is being read for display. If the display of FB1 is finished and FB2 is not yet completely filled, then FB1 is simply displayed again. Once FB2 is filled with the next frame and the display of FB1 is done, a switch occurs; FB2 is now sent off for display while FB1 fills with the next frame. This design allows for display on a monitor with any framerate as long as the pixel clock speeds required for the display are supported. The VGA control module also contains a FIFO that allows for pre-fetching pixels to ensure that pixels are always available and ready for display in case the RAM is busy with other operations. While the VGA PMOD can output 4-bits of R, G, and B, which can produce 2^(4+4+4)^ = 4096 different colours, the camera outputs in greyscale, so the 4 MSBs of each pixel is instead fed into the three RGB channels resulting in only 2^4^ = 16 shades of greyscale on the monitor. Future improvements can include an HDMI output on the custom PCB to avoid the lack of pins required for a VGA display, but VGA was used to ensure compatibility with as many displays as possible.

The main module contains the various combinational logic and finite state machines (FSMs) required to power and configure the image sensor, manage control signals and data flow to facilitate all modules working together, and also handle user input through switches and pushbuttons. The required SPI uploads were stored in order using on-chip block RAM for efficient handling. A reset switch was used as a global reset and to trigger the power down sequence of the image sensor. Switches were also used to boot the camera in pre-configured resolutions and framerates by altering the SPI commands sent, while pushbuttons were used to take pictures, start and stop video capture, and initiate SD card storage, with the LEDs providing feedback on the actions taken. The main module interfaces with the DDR3 (double data rate) DRAM (dynamic random-access memory) through a custom Advanced eXtensible Interface (AXI) design that manually handles the data transfer and handshaking protocols. Although the pixels are captured in 10-bit mode, the pixels are reduced to 8-bits before being stored in DRAM. This is not a limitation in the hardware but instead an intentional trade-off since our specific camera benefitted more from the 25% extra recording time gained as opposed to an increased dynamic range.

The design does not rely on the MicroBlaze soft processor or any IPs requiring purchase, and circumvents the need for the USB3, GigE, and CoaXPress cores that Xilinx advertises in their machine vision designs [28]. The USB3 or GigE links are what some low-cost machine vision cameras from FLIR Inc. utilize, but their connection throughput ultimately hinder the capabilities of the image sensor. For instance, the BFS-U3-13Y3M-C camera uses the USB3 Vision interface with a 380 MB/s maximum throughput limit and the same PYTHON 1300 image sensor as our design, but can only reach 170 fps at maximum resolution compared to the reported 210 fps from the image sensor datasheet, and only up to 663 at lower resolutions [29].

A few IP blocks included with the Vivado webPACK were required to initialize and reset the Zynq PS, as well as generate the required clocks, FIFOs, and memory-mapped registers for communication between the PL and PS. These registers were used to start the transfer of frames to the SD Card for permanent storage. A bare-metal C-application (written in Xilinx SDK 2017.4) that used only one of the ARM cores and did not require Linux stored the captured frames to the SD card using the FatFS library. A small section at the top of the DRAM was reserved to store the application. The pictures were stored as portable grey map (PGM) files, while videos were stored as binary files with the SD card formatted with a large cluster size to maximize transfer speeds. The binary files are then processed on a computer and encoded into videos using the free and open-source FFmpeg tool. The project files including the HDL design, C-application, and PCB Gerber files are available via GitHub (https://github.com/yamnchalich/HFRC.git).

### Nematode experiments

In our tests, nematode *C*. *elegans* were cultured on standard agar-based media plates (LB-Agar) as described previously [30,31]. The strains used in this study are wild-type (N2) and *GFP* fluorescent reporter carrying transgenic animals *myo-3p*::*GFP* and *hsp-4p*::*GFP*. Still and video images of adult animals were captured by the camera mounted on a Zeiss AxioImager D1 Nomarski microscope and Leica MZ-FLIII fluorescent stereomicroscope.

## Results and discussions

### Camera specifications

The configurations implemented are shown in Table 1, utilizing three switches for simple switching between the eight-state combination of resolutions and framerates. Maximum framerates were calculated by measuring the number of clock cycles between Frame Start signals from the sensor to obtain the time per frame and then inverting the value. Although the PYTHON 1300 datasheet reported a maximum of 2235 fps at 256x256, we were able to surpass this. The commonly used framerates of 60 and 30 fps were made available at max resolution, while all resolutions allowed for a roughly halved framerate in exchange for increased exposure in low light conditions. Storage of a full video of roughly 1 GB to the SD card takes approximately 1.5 minutes.

**Table 1:** List of 8 camera configurations supported by 3 PMOD switches.

Resolution	Framerates	Record Time (s) (8-bit pixels, ~1 GB storage)
1280x1024 (SXGA)	211 (MAX)	3.86
	100	8.14
	60	13.57
	30	27.13
640x480 (VGA)	817 (MAX)	4.25
	400	8.69
256x256	2329 (MAX)	6.99
	1000	16.29

This design comes with benefits that may be absent from some commercially available cameras. For instance, the VGA display outputs a continuous live feed from the camera even during recording due to the large DRAM bandwidth and separate VGA frame buffer and storage pipelines. Some cameras, especially those that require a host computer, have to disable the live display during recording. With our addition of PBs and SWs, no host computer is required and the camera can be operated as a standalone device. This removes the need for complicated software that may require training, troubleshooting or maintenance. On top of the standard function to record until the DRAM is full, the option to make the DRAM a cyclic buffer was also implemented. This allowed for continuous recording that captures the past several seconds (depending on resolution and framerate) until stopped–a useful feature for events that are difficult to time. Resource utilization for this design came to ~5% of the available PL resources on the Zynq 7020 SoC, as seen in Table 2.

**Table 2:** Resource utilization of the Zynq 7020 PL for this design.

	Look-Up Tables (LUTs)	Flip-Flops (FF)	Block RAM (# 36 Kb Blocks)
**Used**	2748	3978	9
**Zynq 7020 Pl Total** [26]	53200	106400	140
**Percent Usage**	5.2%	3.7%	6.4%

This helped the camera achieve a low power consumption, with a Tenma 72–7745 multimeter measuring a peak current draw of 0.283 A (3.396 W) at the 6-pin 12 V power connector of the carrier board during recording. Combined with the power dissipation of the custom PCB coming primarily from the image sensor at 0.620 W, this gave the whole camera a power consumption of roughly 4 W, comparable to machine vision cameras in its price range. Additionally, this gives an indication of the relative simplicity of our custom camera design, allowing for more functionality to be implemented or a lower end chip to be used for future implementations that are cheaper and more optimized. A cost breakdown of the camera is detailed in Table 3, with the overall cost of the camera totaling $656 USD at the time of development. It is estimated that a full custom board with only the necessary components of this design can be achieved for one third of this price when considering bulk pricing.

**Table 3:** Cost breakdown of the camera.

Component	Cost (USD)
MicroZed 7020	$213
FMC Carrier Board	$175
Custom Image Sensor PCB	$150
Acrylic cover + C-mount + Spacer	$90
PMODs (VGA + PBs + SWs)	$22 ($9 + $8 + $5)
5 V micro USB power supply	$6
Total:	$656

### Figure-of-Merit (FoM) comparison

To compare our camera against other high-speed cameras on the market, we developed a figure-of-merit (FoM) that takes into consideration important performance parameters of a camera and outputs a single overall performance number, dependent on the framerate and resolution the user is targeting. The equation developed and used was

$$\begin{array}{c} FoM=\frac{\sqrt{RES_{max}}\cdot RT_{RES_{max}}\cdot FPS_{RES_{max}}}{C\cdot P}\cdot \frac{FPS_{max}}{FPS_{RES_{max}}}\\ =\frac{\sqrt{RES_{max}}\cdot RT_{RES_{max}}\cdot FPS_{max}}{C\cdot P} \end{array}$$ (1)

where *RES*_*max*_ is the number of pixels at the maximum resolution for a fixed target framerate, $RT_{RES_{max}}$ and $FPS_{RES_{max}}$ are the recording time (in seconds) and framerate at *RES*_*max*_, respectively, *FPS*_*max*_ is the maximum frames per second for a fixed target resolution, *C* is cost in USD, and *P* the power consumption in Watts. A square root of *RES*_*max*_ was taken to obtain a square dimension and scale the FoM down. The targets set for this comparison was the specifications achieved with our camera: 2329 fps and 256x256 pixels. Thus, the cameras investigated are reported at 2 configurations: the first to determine their maximum resolutions up to and around 2329 fps (if the camera cannot reach the target framerate, the highest reported framerate configuration is used), and again at a configuration as close to 256x256 pixels as possible to determine a maximum achievable framerate. Separating these two targets allows a proper comparison of the cameras’ recording times at a given framerate, as well as give an indication of their high-speed capabilities to offset their cost if they can surpass the target framerate at the target resolution. The separation is also important since the product of the number of pixels with framerate is typically not constant across different configurations of the same camera due to pixel readout architectures and overheads.

The units of the FoM are *N_pixels_*/$ ⋅ *W*, that is, the number of pixels per unit cost and power, where the higher the number, the better is the performance. The time dependence associated with framerates is cancelled out due to including recording times at a fixed framerate. This is almost inverse to a standard metric of Cost/FPS such as in [32], where the lower the number, the better. In comparison, our FoM provides the benefits of the target separation discussed, the inclusion of recording times for frame buffering capabilities, as well as power consideration. While cooling strategies may be reflected in the cost of some commercially available cameras, power considerations are still valuable in indirectly determining the bulkiness, reliability, and potential for portability with sustained battery-powered operation. We have nonetheless included an equivalent Cost/FPS metric for comparison, with frames normalized to a 256x256 resolution using the FPS_max_ values. Table 4 contains the FoM and Cost/FPS comparisons between our camera, some machine vision cameras and some of the best affordable standalone cameras with publicly available specifications and prices. These prices are also subject to change with time.

**Table 4:** FoM comparison of several low-cost, high-speed cameras against our prototype.

Category	Camera	RES_max_ @ FPS (up to ~2329 fps)	Record Time (s) (8-bit pixels)	FPS_max_ @ RES (down to ~256x256)	Power (W)	Cost (USD)	FoM (N_pixels_/ $⋅W)	Cost/FPS (256x256 frames) ($⋅s/frame)
Stand-alone	Chronos 1.4 [9]	1024x576 @ 2359	6.17	15200 @ 336x252	19.2^a^	2999	1251	0.15
	fps4000 Lite [10]	1280x720 @ 950	60	950 @ 1280x720	11.1^b^	2421^c^	2036	0.18
	Edgertronic SC1 [33]	640x480 @ 2324	22.41^d^	7181 @ 320x240	30	5495	541	0.65
	Sony RX100 V [34]	912x308 @ 1000	7	1000 @ 912x308	2.6	1000	1427	0.23
Machine Vision	GS3-U3-23S6M-C [35]	416x240 @ 697	1.84^d^	697 @ 416x240	4.5	1045	86	0.98
	BFS-U3-04S2M-CS [36]	320x240 @ 997	3.13^d^	997 @ 320x240	3	365	791	0.31
	BFS-U3-13Y3M-C [37]	320x240 @ 663	4.71^d^	663 @ 320x240	3	415	696	0.53
	FL3-GE-13S2C-CS [38]	320x240 @ 136	3.06^d^	136 @ 320x240	2.5	695	66	4.36
	Our Prototype Camera	256x256 @ 2329	6.99	2329 @ 256x256	4	656/751^e^	1588/1387^e^	0.28/0.32^e^

^a^ Assuming EN-EL4a (28.9 W⋅h) batteries for the reported 1.5 hours battery life when recording.

^b^ Assuming 2 x 18650 Li ion (3.7 V, 3000 mA⋅h) batteries for the max reported 2 hours battery life.

^c^ Converted from Pound sterling.

^d^ Calculated using internal buffer at RES_max_ config. (from top to bottom: 16 GB, 128 MB, 240MB, 240 MB, 32 MB).

^e^ These values consider the inclusion of lens ($95) for standard camera operation.

Although resolutions close to 256x256 was chosen for *FPS*_*max*_, the fps4000 Lite comes with a fixed configuration of 720p, potentially skewing its FoM. For the battery powered and portable Chronos 1.4 and fps4000 Lite cameras, the power was calculated by researching the typical W⋅h rating of the battery and using the maximum reported usage/recording time for the camera. The machine vision cameras can record for much longer periods thanks to their reduced framerates and dependence on the memory of a host computer, but for proper inclusion to the FoM comparison against standalone cameras, the reported internal buffers of the machine vision cameras were used to calculate a theoretical recording time.

While the above comparison is based on pure standalone hardware performance and high-speed functionality for the price, it is important to consider the extra developmental costs associated with building a camera as opposed to purchasing one. Development of this prototype from scratch took roughly 4 man-months with little prior experience in hardware programming and PCB design and required soldering experience. While others can now replicate all or parts of this work to significantly reduce their own developmental efforts, the “plug-and-play” nature of commercial cameras may still be more suitable for some. However, since commercial systems do not allow modification of its hardware or codebase, those in need of a platform in which they can add unique functionality or test custom image processing techniques and algorithms can benefit greatly from an open-source platform with direct access to an image sensor pixel stream. This user-customizable, high-speed camera also has value as an educational tool for students and researchers considering its relatively low cost.

### Camera customizability

**Fig 4:**
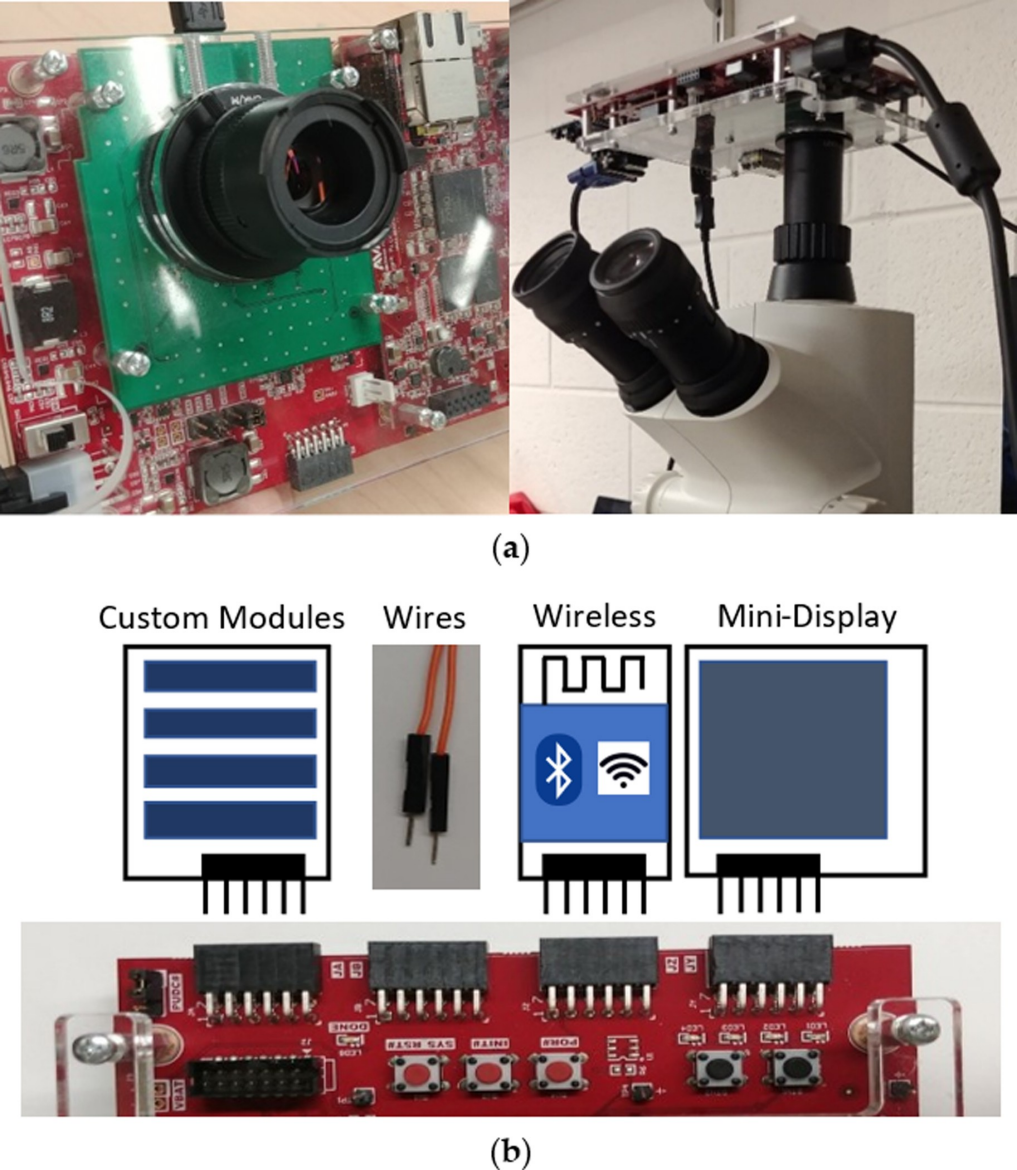
Examples of operation and some hardware customizability examples. (a) Camera with attachable lenses or mounted onto a microscope; (b) Different modular attachments or custom solutions to fit the user’s needs.

Various aspects of the design and build of our camera system allow for modifications that can range from simple to complex. This includes adding software or hardware code to replacing or creating custom PMOD attachments and boards. Currently, the camera contains a C-mount for attachable lenses and mounting on microscope systems, along with PMOD VGA output, pushbuttons, and switches. These can be substituted for different modular attachments that are commercially available, such as the Bluetooth or 96x64 RGB OLED (organic light-emitting diode) display PMODs from Digilent Inc. [39]. Example code and resources are provided for them online and they give the added functionality to control the camera remotely or offer a miniature real-time display, omitting the need of a monitor. Custom attachments are also viable, and any remaining or exposed pins can be manually connected to. These types of hardware modifications are illustrated in Fig 4.

More complex modifications can include the addition of an HDMI output on the custom PCB along with a custom HDMI Verilog module to bypass the bit-depth limitation of the VGA output. Additionally, should higher framerates or higher resolutions be needed for an application, more demanding image sensors can be substituted given the 36 LVDS pairs that the FMC connection allows for. The PCB and Verilog code would have to be modified to handle the extra data lines, but the overall cost of the camera would primarily rise only with respect to the image sensor. With the MicroZed Zynq SoCs having up to 24 LVDS pairs per I/O bank capable of 950 Mb/s of DDR data and a DRAM bandwidth above 4 GB/s (1066 MT/s on an 32-bit wide interface) [40], custom carrier boards or fully custom board designs can maximize I/O usage and minimize size, power, and cost.

Regarding the codebase, extra software can be written on the processor for a more sophisticated user experience, and the USB and Ethernet ports allows the camera to communicate with a host computer if preferred. However, care is to be taken how much memory is required for the application should it have to be stored in DRAM, affecting the recording length. Moreover, the current very low utilization of the programmable logic means a wide range of processing capabilities can also be implemented. With FPGA circuits being highly parallel and spatially oriented on the fabric, extra Verilog modules can be added to the code base with little to no impact on existing performance as long as the signals and data are properly buffered to meet timing requirements.

Simple features that may be difficult to find in commercial cameras are readily implementable due to the user having access to all the internal signals in the Verilog code. For instance, individual frames of a video can be tagged during recording using input pulses to mark specific events, proving useful in synchronized testing environments involving other sensors. Accessing the frame synchronization information from the image sensor in Verilog and reading an input pulse from a wire on a spare PMOD pin (at the appropriate voltage level of the carrier board) makes this relatively easy to implement. The large remaining resources can be utilized to create and test custom image processing algorithms such as real-time image classification [41] and object detection [42]. Some of this research requires separate high-speed and potentially expensive cameras as input, such as in [43] where two high-speed camera heads are used with a platform consisting of two FPGAs to create a high-speed vision system. The same team also described a fast multi-object feature extraction algorithm [44] implemented on one of the FPGAs with low resource usage capable of fitting within the remaining PL resources from our design. A system based around our camera design provides a cost-effective platform with customizability suitable for building and testing such systems and algorithms.

## Application

### General applications

A convenient sampling rate or framerate for an event is typically several times the event frequency, but can be lower as long as it is greater than twice the event frequency required by the Nyquist Sampling Theorem [7]. Thus, cameras that can achieve framerates into the low thousands are useful in a wide range of applications that require up to millisecond or sub-millisecond time resolutions. A recent proposal for a low-cost test set-up for impact experiments on dummy heads suggested framerates equal to or above 200 fps [2]. Tests were also done on the free-fall drop impact of portable products at 1000 fps [3] and automotive crash testing cameras typically run between 1000–4000 fps to meet automotive safety compliance standards [45]. Furthermore, such cameras can be used for biomechanics research, such as was done with the analysis of wing flap aerodynamics of the hummingbird [46] as well as their comparison to that of bats [47], with recordings from 500–2000 fps. Research has also investigated the relationship between trans-glottal airflow and vocal fold vibration (recorded at 1900 fps at 256x64 resolution) [48], with more recent work analyzing the effect of resolution on the laryngeal parameters computed on the captured glottal area waveform (4000 fps, resolutions at or below 512x256) [49]. Even a study in delay eyeblink conditioning in monkeys [50] had required high-speed video measurements at 2421 fps and 128x128 pixels, a configuration similar to what our camera can achieve. Moreover, popular technology reviewers use high-speed imaging to perform comparisons or verify commercial product claims. One such test compared the input lag associated with Nvidia’s G-Sync and AMD’s FreeSync technologies using a Sony FS700 camera at 960 fps [51] since the propagation of a signal from keyboard to monitor is on the order of milliseconds.

Low-cost high-speed camera systems are suitable for many of these applications and more since they do not require high-end equipment or features and can be run at reduced resolutions. In addition, the cost benefit of such cameras becomes important when considering applications that may currently be done using other sensor technologies. An example of this is the measurement of human reaction time, which can be difficult to accurately and inexpensively measure. While low-cost techniques have been developed to tackle this such as with wireless motion sensors [52], the use of a low-cost high-speed camera remains a simple and effective solution capable of providing higher accuracy to either replace, test or calibrate existing systems.

**Fig 5:**
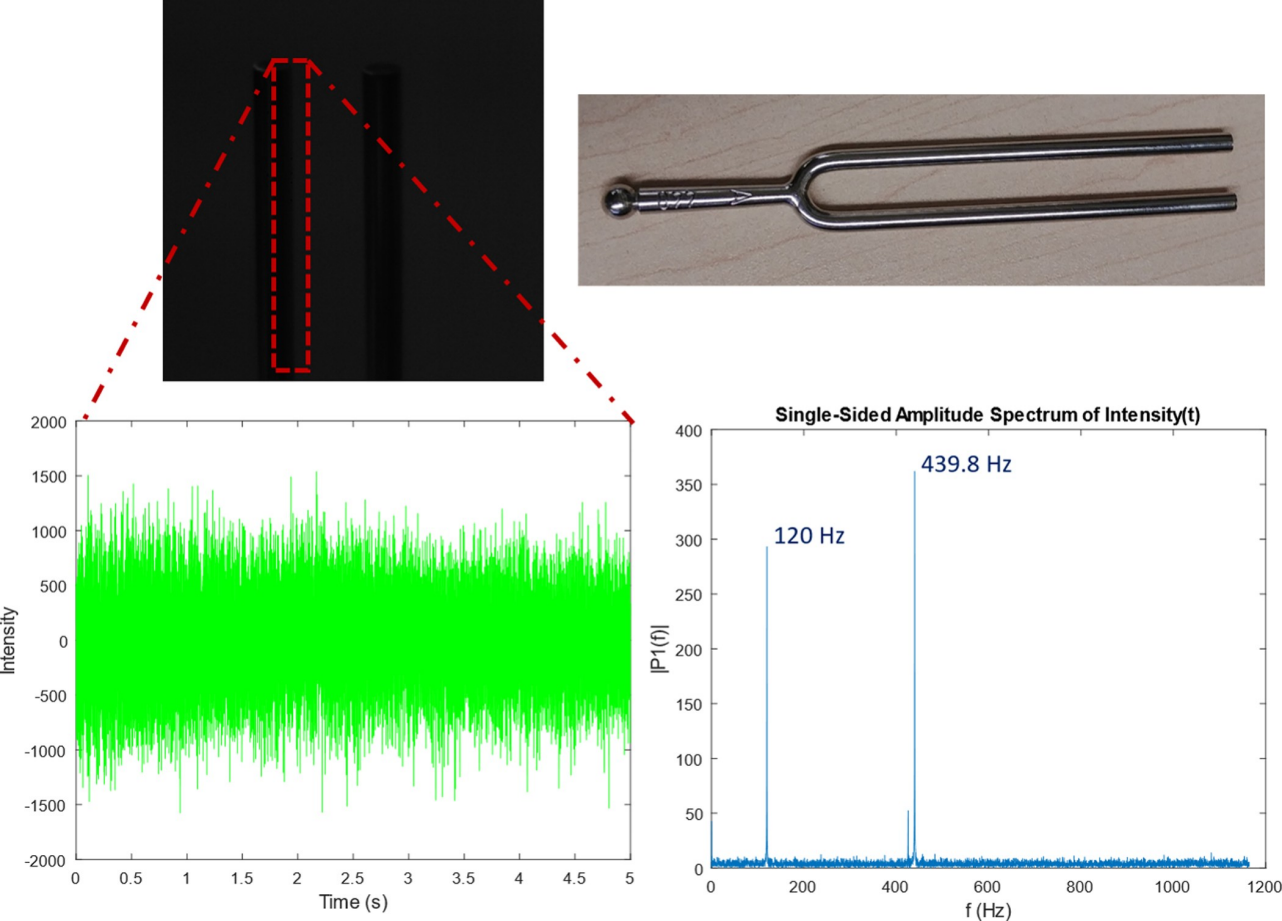
A 440 Hz tuning fork captured at 2329 fps using only ambient lighting. Fourier analysis identified the fork’s frequency as well as the 120 Hz flicker of the ambient fluorescent lighting.

A demonstration of the camera’s high-speed capabilities is in recording sound vibrations, akin to research from [53] that used high-speed cameras to “image sound”. Tests were run at 2200 fps at 700x400 resolution using a Phantom V10 camera to observe the low frequency spatial vibrations of objects and obtain speech and sound from the resulting high-speed video. One important limitation of this technique is the use of expensive cameras to record video at high enough framerates to cover a suitable range of frequencies. We demonstrate capturing the vibrations of an inexpensive 440 Hz tuning fork at 2329 fps with 256x256 resolution. Its frequency, as well as the 120 Hz flicker from the ambient fluorescent lighting, was determined accurately from an FFT of the changes in pixel intensity of a cropped section of the frames focused on the vibration of one fork tine, as seen in Fig 5. The experiment was run with no extra light sources resulting in dark frames due to the low exposure associated with such a high framerate. This was done to test the success of such an experiment without the need of investing in extra lighting equipment.

A key research field where such cameras can be most useful is in microscopy. Here, strong sources of light usually accompany microscope systems, creating an environment that is well-suited for high-speed capture. Depending on the wavelength (*λ*) of light and the numerical aperture (*NA*) of the objective and condenser lenses used, the optical resolution (*r*_*opt*_) achievable by a microscope is given by the Rayleigh criterion [54] as

$$r_{opt}=\frac{1.22\lambda }{NA_{obj}+NA_{cond}}$$ (2)

and represents the minimum resolvable distance. Assuming green light (*λ* = 525 nm) and a microscope system with *NA*_*obj*_ of 0.65 and *NA*_*cond*_ of 0.95, the optical resolution would be ~0.4 μm. With a magnification (*M*) of the objective lens and a target sampling rate within the optical resolution obtained from Eq 2, the pixel size is calculated as

$$pixel\;size=\frac{M\cdot r_{opt}}{sampling\;rate}$$ (3)

Three to six pixels within the optical resolution was shown to be ideal for cell microscopy [55–57], satisfying the Nyquist Sampling Theorem without excessive oversampling that can create false details due to secondary diffraction. Thus, using the optical resolution of 0.4 μm previously calculated, along with a magnification of 10x and a target sampling rate of 3, a required pixel size of ~5.3 μm can be calculated. The small 4.4 μm pixel size of the PYTHON 1300 image sensor selected for our camera makes it suitable for this example and other common microscope configurations.

### Application involving a nematode system

**Fig 6:**
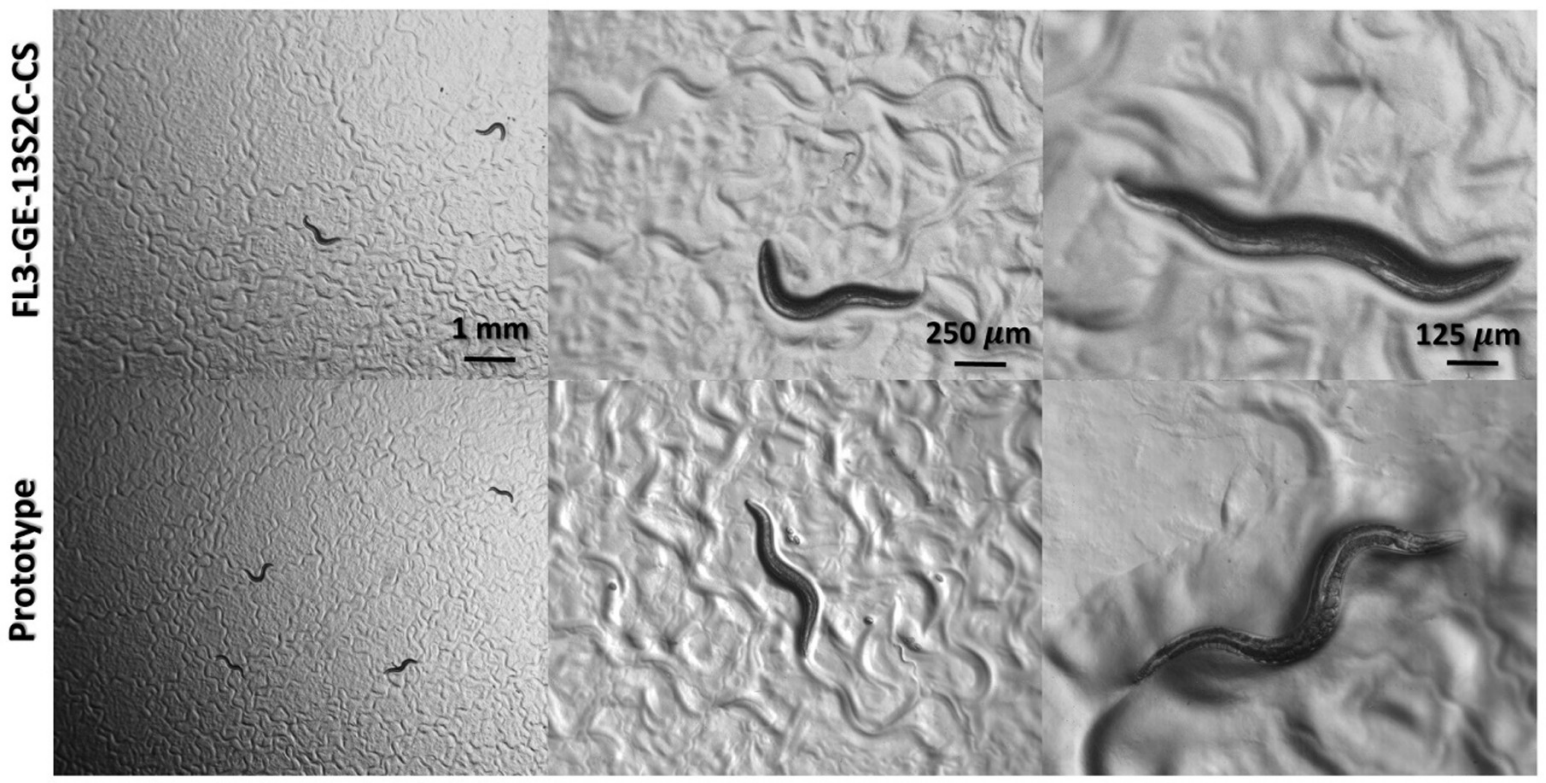
A comparsion of images at 3 different magnifications using the FL3-GE 13S2C-CS (top row) and our prototype (bottom row) at their maximum resolutions (1288x964 and 1280x1024, respectively) and 30 fps for both. All the images show day-1 wild type adult animals.

Our camera was compared against an existing FL3-GE 13S2C-C machine vision colour camera (included in the FoM table) currently in use as a microscope camera to test single image quality of *C*. *elegans*. *C*. *elegans* (commonly referred as nematode or worm) is the leading animal model to study the conserved biological pathways and disease phenomena in a laboratory setting [58,59]. The animals are transparent with adults of about 1 mm length that feed on microbes, primarily bacteria. Since their first introduction by Sydney Brenner [30], *C*. *elegans* have been used extensively in biomedical research in wide areas such as development, neurobiology, cell biology, and aging. Among other advantages, these animals offer a short life cycle, small size, compact genome and the ease of propagation. Fig 6 shows a few frames taken with both cameras at their native resolutions and at 30 fps for comparable results. Our camera shows similar or better image quality for a lower cost, while being standalone and customizable. This gives it the added benefit of unhindered high-speed capture, real-time display even during recording, continuous recording with a cyclic frame buffer, and no need for a host computer.

**Fig 7:**
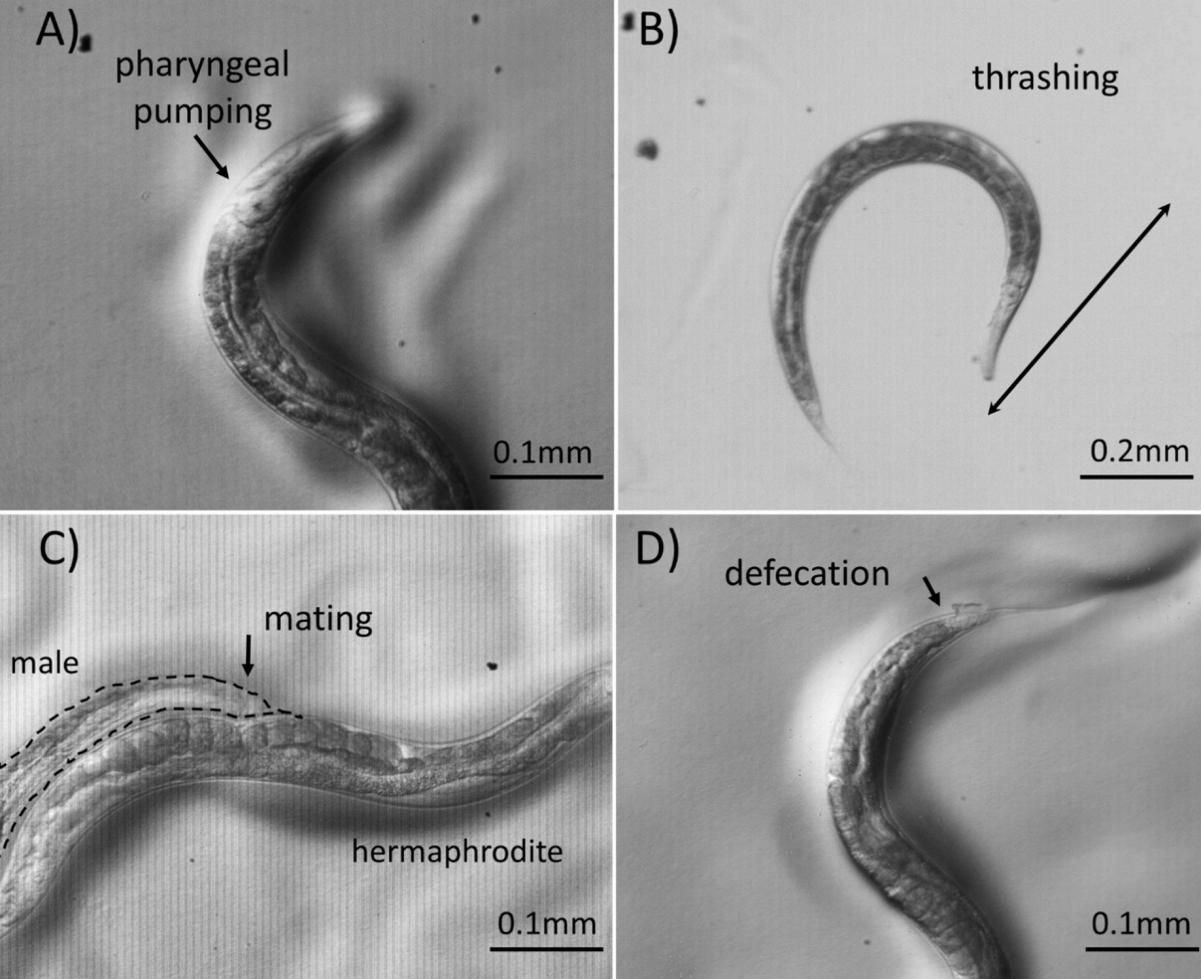
Aging-associated phenotypes and other nematode specific behavior are made easier to analyze by capturing videos at high speed. Still images of various processes taken from the videos of respective speed are included: (a) pharyngeal pumping at 100 fps; (b) thrashing at 210fps; (c) mating behavior at 210fps and 8x analog gain; and (d) defecation at 210fps.

*C*. *elegans* has been used extensively for research on aging related processes. The hermaphrodites have a short lifespan of around 3 weeks making it possible to study age-associated changes in tissues and processes [60]. Genetic experiments in *C*. *elegans* have uncovered the roles of many aging-associated genes and pathways that are conserved across eukaryotes [61,62]. The findings have revealed that aging is a progressive increase in fragility that results in increased mortality rate as a function of time [63]. Two of the age associated markers in *C*. *elegans* are body bending and pharyngeal pumping (i.e., feeding). The rates of these physiological markers decline as animals get older. However, since the existing procedures typically involve manual scoring of these changes [64], the quantification is labor intensive, subjective, and prone to error. Furthermore, alterations in phenotypes cannot always be reliably detected. Our camera allows the high-speed observation and recording of subtle changes during aging of animals (see some examples in Fig 7 and S1 Movie). The camera is also capable of capturing various other behavioral processes such as defects in mating, thrashing rate, defecation length cycle and real time rolling (Fig 7 and S3–S5 Movies).

**Fig 8:**
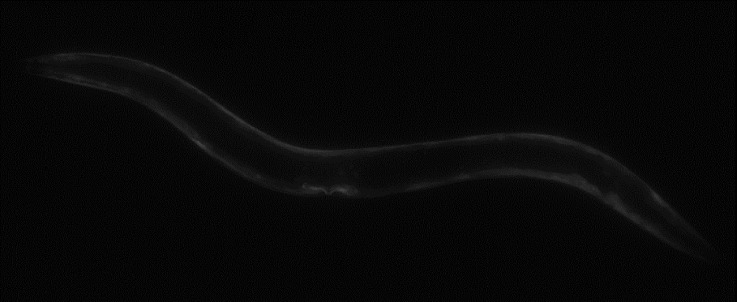
*GFP* fluorescence captured at 30 fps. Image composed of two pictures stitched together to obtain a wider view. Transgenic animal expressing *myo-3p*::*GFP* has been used.

While this high-speed imaging and recording is possible due to strong microscope light sources, the imaging of fluorescence, for instance, is difficult at high framerates due to the potentially very low fluorescent light levels. These situations favour large pixel sizes for better light collection, especially if trying to record at high framerates, putting the small (4.4 μm) pixel size of the image sensor used in our camera at a disadvantage. By recording at higher exposures (reduced framerates down to 30 fps) or modifying the digital or analog gain (at the cost of potential noise increase), it becomes possible. This is demonstrated in Fig 8, where a *GFP*-tagged *C*. *elegans* worm was imaged with increased exposure at 30 fps with good quality. However, a vertical striping pattern becomes apparent in low light and increased gain conditions which is believed to either be a consequence of the electrical characteristics of the electronic circuitry associated with each pixel and column output amplifier [14] or due to imperfections in the image sensor power supplies and readout electronics as a similar effect was observed in the testing of the Chronos 1.4 [20]. This is slightly observable in Fig 7 with vertical line patterns, particularly in Fig 7C) which was taken at 8x analog gain. Similarly, a video of the fluorescent worms was also captured at 100 fps with 8x analog gain (see S6 Movie) where the noise pattern affects the resulting quality. Further work is required to assess the source of the noise and expand the codebase with image processing modules to allow the design of a more robust and reliable system in future iterations.

## Conclusions

We developed a low-cost, standalone, high-speed camera prototype capable of >2000 fps at reduced resolutions using a Zynq System-on-Chip development environment and a custom, open-source hardware descriptive language design. The camera was shown to be competitive to some commercial standalone and machine vision high-speed cameras using a developed Figure-of-Merit showing their price-to-performance values. Our camera avoided the hardware restrictions that hindered low-cost machine vision cameras while offering similar functionality to more expensive standalone cameras. The customizability of the camera as a result of the low programmable logic resource usage and potential for custom or commercial modular attachments is explored, providing a cost-effective platform for the development and test of high-speed imaging systems and image processing algorithms. The camera proved successful in the capture of low frequency spatial vibrations in low light conditions and was tested against an existing microscope camera, revealing greater details of processes used to investigate age-associated physiological changes in *C*. *elegans*. Future work is expected to reduce the size and cost of the camera while greatly expanding on its processing capabilities using the remaining programmable logic resources in order to create a more easily replicable and convincing alternative to commercial systems.

## Supporting information

S1 Movie Pharyngeal pumping (100 fps video slowed to 30 fps). Uncompressed AVI file can be found at https://github.com/yamnchalich/HFRC.git.
(M4V)

S2 Movie Thrashing (211 fps video slowed to 30 fps). Uncompressed AVI file can be found at https://github.com/yamnchalich/HFRC.git.
(MP4)

S3 Movie Mating behaviour (211 fps video at 8x analog gain slowed to 30 fps). Uncompressed AVI file can be found at https://github.com/yamnchalich/HFRC.git.
(MP4)

S4 Movie Defecation (211 fps video slowed to 30 fps). Uncompressed AVI file can be found at https://github.com/yamnchalich/HFRC.git.
(MP4)

S5 Movie Rolling phenotype (211 fps video slowed to 30 fps). Uncompressed AVI file can be found at https://github.com/yamnchalich/HFRC.git.
(MP4)

S6 Movie GFP fluorescent animals in the liquid medium. Transgenic animals expressing *hsp-4p*::*GFP* have been used (100 fps video at 8x analog gain slowed to 30 fps). (MP4) Uncompressed AVI file can be found at https://github.com/yamnchalich/HFRC.git.
(MP4)
